# Color naming deficits and attention-deficit/hyperactivity disorder: A retinal dopaminergic hypothesis

**DOI:** 10.1186/1744-9081-2-4

**Published:** 2006-01-27

**Authors:** Rosemary Tannock, Tobias Banaschewski, David Gold

**Affiliations:** 1 The University of Toronto, Toronto, Canada: Brain and Behaviour Research Program, The Hospital for Sick Children, Toronto, Canada; 2 Child and Adolescent Psychiatry, University of Göttingen, Germany; 3 Centre for Advanced Study at the Norwegian Academy of Science and Letters 2004–2005, Norway; 4 The University of Western Ontario, London, Canada

## Abstract

**Background:**

Individuals with Attention-Deficit/Hyperactive Disorder (ADHD) have unexplained difficulties on tasks requiring speeded processing of colored stimuli. Color vision mechanisms, particularly short-wavelength (blue-yellow) pathways, are highly sensitive to various diseases, toxins and drugs that alter dopaminergic neurotransmission. Thus, slow color processing might reflect subtle impairments in the perceptual encoding stage of stimulus color, which arise from hypodopaminergic functioning.

**Presentation of hypotheses:**

1) Color perception of blue-yellow (but not red-green) stimuli is impaired in ADHD as a result of deficient retinal dopamine; 2) Impairments in the blue-yellow color mechanism in ADHD contribute to poor performance on speeded color naming tasks that include a substantial proportion of blue-yellow stimuli; and 3) Methylphenidate increases central dopamine and is also believed to increase retinal dopamine, thereby normalizing blue-yellow color perception, which in turn improves performance on the speeded color naming tasks.

**Testing the hypothesis:**

Requires three approaches, including:1) direct assessment of color perception in individuals with ADHD to determine whether blue-yellow color perception is selectively impaired; 2) determination of relationship between performance on neuropsychological tasks requiring speeded color processing and color perception; and 3) randomized, controlled pharmacological intervention with stimulant medication to examine the effects of enhancing central dopamine on color perception and task performance

**Implications of hypothesis:**

If substantiated, the findings of color perception problems would necessitate a re-consideration of current neuropsychological models of attention-deficit/hyperactivity disorder, guide psycho-education, academic instruction, and require consideration of stimulus color in many of the widely used neuropsychological tests.

## Background

Attention-Deficit/Hyperactivity Disorder (ADHD) is a common and impairing childhood-onset disorder characterized by inattention, hyperactivity, and impulsiveness that affects 3–7% of school-aged children and tends to persist into adulthood [1]. Converging neuroscience evidence suggests that ADHD is often associated with impaired executive functioning, probably arising from widespread alterations of neuronal circuits including the prefrontal cortex, basal ganglia, parietal cortex, anterior cingulate, and cerebellum. Hypodopaminergic neurotransmitter functioning is believed to play a central role in its pathophysiology [2-5]. Although sensory and perceptual abilities have been presumed to be intact in ADHD [2,4,5], recent findings are challenging this earlier assumption. For example, reduced visual perceptual sensitivity has been demonstrated in children with ADHD [6,7]. Also, individuals with ADHD exhibit unexplained problems in the speeded processing of colored stimuli [8-22], and stimulant medication, (a common treatment approach for ADHD) is reported to selectively improve naming speed for colors but not for other types of stimuli [8,23].

To date, there is no adequate explanation to account for either the observed color processing problems in ADHD or their selective amelioration by psychostimulant medication. We propose that the color processing problems may reflect subtle detrimental effects of dopaminergic deficiency in the central nervous system on retinal dopamine, which in turn impedes the efficiency of the short-wavelength (blue-cone) color vision mechanism as well as of other visual functions. Prior to elaborating on this hypothesis, we first review the evidence of problems in color processing associated with ADHD and the existing explanatory accounts. We then outline possible empirical approaches to test this hypothesis, and discuss the theoretical and clinical significance of the hypothesis should it be substantiated.

### Color processing problems in ADHD

Several lines of evidence indicate impaired performance on tasks requiring rapid and/or continuous processing of colored stimuli in ADHD. For example, in a seminal study on rapid automatized naming speed, Denckla [9] described four of the five boys with dyslexia who exhibited slow color naming as being "inattentive", but not "hyperkinetic". Notably, most of the boys' errors were associated with naming the colors blue and yellow, but tests for color blindness revealed no abnormalities [9]. Subsequent studies of rapid naming have found consistently that children and adolescents with ADHD exhibit slower naming speed for colors on the Rapid Automatized Naming Test and Stroop Color Word Test, but typically do not exhibit slower naming of letters, words, or digits [8,10-22]. Slower color naming on the Stroop Color-Word Test is sometimes found even in the absence of slower word naming or poor interference control [19]. ADHD is also associated with taking more trials to deduce the first sorting rule on the Wisconsin Card Sorting Task [13,24]: the first rule requires participants to sort according to the color of the stimuli and ignore their shape and number. Moreover, one study demonstrated that the number of trials taken to deduce the first sorting rule was associated with slower Stroop color naming in the ADHD group but not in the control group, suggesting that color processing deficits may underlie performance on both tasks [13]. Furthermore, children with ADHD have been found to respond significantly more slowly (about 200 ms slower) to blue stimulus shapes compared to normal peers, but do not differ in their response time to green stimuli in a visual processing task [22]. Collectively, these findings suggest impairments in color perception and preliminary support for this proposition is provided by a recent study of selective attention to color using event-related potentials. The findings indicated that boys with ADHD exhibit an early *perceptual deficit *in selection of visual stimuli on the basis of *color *(red versus blue) as well as in later semantic stages of visual selective attention [7].

### Current explanations of poor color processing in ADHD

Currently, two classes of explanation (psychological, neurobiological) are discernible in the literature for the observed problems in rapid processing of colored stimuli in individuals with ADHD. These theoretical accounts are not mutually exclusive, but rather they overlap and reflect perspectives taken from different research approaches and different levels of measurement.

Psychological accounts attribute slow color naming to developmental immaturity. For example, the development of color naming appears to be much more difficult for young children than naming shapes or animals [25-27]. Also, whereas the fastest speeds of naming alphanumeric stimuli are reached by the age of 16, the speed of naming colors and objects continues to improve (i.e., become faster) into mature adulthood [28]. Accordingly, the overall immaturity in development associated with ADHD might exacerbate the normal developmental lag in rapid naming of stimulus colors. An alternative psychological explanation attributes slow color naming to developmental problems in effortful semantic processing, which is typically associated with right hemisphere function [8]. Specifically, naming of colors (as well as naming of natural objects) is thought to require more effortful, perceptual and/or semantic processing than naming letters, digits, or man-made objects [30-34]. For instance, terms such as digits, letters, and shapes refer to categories with sharp, clear, and non-overlapping boundaries, while color terms refer to categories with unclear, variable, and overlapping boundaries. Often, there may be more than one plausible name for a given color and asymmetries likely exist between the candidate names (e.g., they may differ in word frequency), thus giving rise to response competition and necessitating more careful and detailed processing or the need to inhibit the more frequent or salient color name [6,25]. A related explanation based on response-competition was also proposed by Brodeur and colleagues [22] to account for slower processing of blue stimuli compared to green stimuli. Specifically, this finding was attributed to developmental problems in set shifting from the predominant response to the more common green stimuli than to the non-dominant response to the less frequent blue stimuli. Impairments in semantic processing, set-shifting, and response-competition, have all been implicated in ADHD [7,14,35].

An existing neurobiological account of color processing problems in ADHD associates poor color naming or poor color processing performance to alterations in the underlying neural substrate. For example, slower RAN color naming speed (but not the naming speed for RAN letters, objects or digits) was found to be related to smaller anterior superior white matter volumes (both right and left hemispheres) in ADHD compared to healthy controls [29]. Decreased total white matter volumes in children and adolescents with ADHD, which have been reported in several studies [36-39], suggests delayed or deviant myelination of major fiber tracts such as the corpus callosum, which might have a detrimental impact on the speed of interhemispheric transmission involved in speeded color naming.

Current explanations referring to poor sustained attention, problems in set shifting or semantic processing, or differences in white matter volumes associated with ADHD can not easily account for the dissociation between rapid naming of colored stimuli and rapid naming of letters, digits, or words. Nor can they adequately account for the indications that color processing problems appear to be greater for yellow and blue stimuli. Moreover, from a developmental perspective, the neurodevelopmental immaturity hypothesis would predict greater problems in rapid color naming in children with ADHD compared to adolescents, with possible attenuation of the deficits in adults with ADHD. To date this hypothesis has not been tested directly, but meta-analyses of cognitive deficits in children, adolescents, and young adults with ADHD indicate color naming deficits of moderate to large effect size (d = .58–.62) across the life span with no evidence of age-related changes [19,40].

## Presentation of the hypothesis

Color perception is controlled, at least in part, by retinal dopaminergic neurons [41]. We propose that slowed color processing and color naming in ADHD reflects a specific problem in blue-yellow color perception, which arises from hypo-functioning retinal dopaminergic mechanisms. In the absence of any evidence to the contrary, changes in central and retinal dopamine are believed to occur together. Thus, hypofunctioning of the central dopaminergic system associated with ADHD [4] will be accompanied by hypo-functioning retinal dopamine. The abnormalities in retinal dopaminergic tone will give rise to subtle, but detrimental effects on the on several aspects of visual function, particularly on the short-wavelength chromatic pathway that is responsible for blue-yellow color perception. Normalization of central dopaminergic functioning via pharmacological intervention with psychostimulant medication will normalize retinal dopamine, which in turn will normalize blue-yellow color perception and performance on tasks requiring speeded color naming.

To present the detailed hypothesis, we first provide the necessary background on color perception and the role of retinal dopamine, then argue how hypo-dopaminergic functioning in ADHD will give rise to impairments in the tritan color mechanism, which in turn will influence speeded color naming on tasks involving a substantial proportion of blue and yellow stimuli.

### Color perception and the role of retinal dopamine

Color perception is based on the three cone photoreceptor types maximally sensitive to long, middle, and short wavelengths in the perceived light spectrum that constitute two functionally and anatomically distinct systems at the retina and lateral geniculate nucleus; a 'red-green' system with a foveal specialization in which long and middle wavelength cone signals are antagonistic, and a 'blue-yellow' pathway in which short wavelength cones are opposed by a combined signals from long and middle wavelength cones without such a foveal overrepresentation [42]. Normal development of the blue-yellow (tritan) color mechanism appears to lag behind that of the red-green mechanism, which is functional in human infants by 2 months of age [43-46].

Most color perception defects (i.e., dyschromatopsias or 'color blindness') are congenital and arise from altered sensitivity defects of the L and M cones. By contrast to these red-green color vision deficits, which respectively affect 2% and 6% of the male population, congenital defects in S-cone sensitivity are rare (about 0.01%) but affect both sexes equally [47]. Acquired dyschromatopsias arising from exposure to environmental pollutants [48] usually impair blue-yellow color discrimination. For example, pronounced effects on color perception, often dose-dependent and involving the short-wavelength (blue-yellow) mechanism, are reported following both acute and chronic exposure to organic solvents and elemental mercury [49,50]. Moreover, occupational exposure to organic solvents during pregnancy is associated with increased risk of color vision and acuity impairment in the offspring [51,52].

Color perception problems, particularly involving the blue-yellow (tritan) mechanism, have also been linked with alterations in retinal dopamine: dopamine is a major neurotransmitter in the mammalian retina [41,53]. Dopamine receptors, DRD1 and DRD4 that seems to be associated with a subsensitive postsynaptic receptor if coded by the 7-repeat allele [54] are both found in the retina [53-55]. Retinal dopaminergic neurons are involved in controlling the coupling of horizontal and amacrine cell lateral systems, the organization of the ganglion cell and the bipolar cell receptive fields and modulation of the physiological activity of photoreceptors. Thus, the retinal dopamine system influences light adaptation as well as other visual functions, including color perception, contrast sensitivity, and spatial and temporal processing [41,53]. The hypothesis proposed focuses on the role of retinal dopamine in color perception.

Alterations in the level of retinal dopamine are reflected particularly in deficits in the short-wavelength chromatic pathway that is responsible for blue-yellow color discrimination, a system that appears to be especially vulnerable to the effects of disorders and drugs [41,50,56-58]. For example, discrimination along the blue-yellow axis (compared to the red-green axis) is particularly impaired in various disorders involving altered dopaminergic mechanisms. Thus, specific blue-yellow color vision disturbances are found in Tourette Syndrome [59], Parkinson's disease [60-63], and Huntington's disease [64]. Changes of retinal dopamine levels arising from cocaine-withdrawal [65-67] and normal aging [41,68] have also been associated with blue-yellow color vision losses.

The fundamental mechanisms causing a specific retinal impairment of color discrimination along the blue/yellow axis in dopaminergic disorders and acquired dyschromatopsias remain unclear. Short wavelength sensitive cones may be more fragile than long and medium wavelength sensitive cones or their relative scarcity and anatomical distribution may be responsible for the greater vulnerability of the blue-yellow perception by alterations of the dopaminergic system [41,47,53,69]. Accordingly, abnormalities in dopamine production, transport, uptake, or receptor sensitivity could result in impairments in visual processing including color – particularly the color blue. Moreover a selective impairment of the blue-yellow vision system suggests a retinal location of the disturbance rather than a central one [41,47].

### Hypo-dopaminergic functioning in ADHD influences color perception

Hypo-dopaminergic functioning has been postulated in ADHD [4,70] and abnormal levels and density of the dopamine transporter in the brain have been reported in adults with ADHD [71]. Moreover, ADHD has been associated with anomalous alleles of the D1 and DRD4 receptors [72-74]. Thus, dopaminergically-related impairments in visual functioning, and particularly in blue-yellow color perception, are plausible in ADHD.

We propose that the observed impairments in individuals with ADHD on tasks requiring speeded color processing of blue-yellow stimuli might be attributable in part to hypo-functioning of both central and retinal dopamine. Critical to this hypothesis is the premise that alterations in central and retinal dopamine occur in parallel (and that pharmacologically-induced increases in central dopamine also increases retinal dopamine). To our knowledge there is no direct evidence that this is the case. Rather, we draw inferences from the following evidence: 1) cerebrospinal concentrations of a metabolite of CNS dopamine, homovanillic acid, correlates positively with electroretinogram blue-cone amplitude [67]; 2) in Parkinson's disease, death of dopaminergic neurons in the CNS also extend to the retina, resulting in impaired visual functions including blue-yellow color perception [61-63]; 3) the visual deficits in Parkinson's disease are mostly reversed by treatment with the dopamine precursor L-DOPA [41]; 4) methylphenidate, which is the primary treatment modality for ADHD, blocks the re-uptake mechanism of the dopamine transporter, increasing the amount of extracellular dopamine able to bind to its receptors [75]. Thus, changes in the dopamine system, regardless of whether brought about experimentally (lesions, pharmacologically) or naturally (as in ageing or in clinical conditions), leads to predictable changes in retinal function [41,53].

According to this retinal dopaminergic hypothesis, deficits in blue-yellow color perception result in poor performance on many of the standard neuropsychological tasks (e.g., Stroop, RAN, Wisconsin Card Sorting Task) that include a substantial proportion of blue-yellow stimuli. Thus poor task performance may reflect subtle impairments in color vision as well as, or instead of, impairments in higher-order cognitive function. Consistent with this prediction are the recent findings that individual differences in color perception influence performance on the classic Stroop color-word task and that incongruent opponent color pairs (e.g., the word *BLUE *in yellow ink) decrease the strength of Stroop interference compared to non-opponent color pairs (e.g., *BLUE *in red ink) [76]. A neural network simulation of the data confirmed that the difference in magnitude of Stroop interference between incongruent color-word pairs involving opponent versus nonopponent colors was attributable to sensory processing of the physical color of the stimuli (particularly the color yellow), which occurs at the level of retinal ganglion cells [76]. Also, it has been demonstrated that visual function deficiencies associated with normal aging (reduced acuity, contrast sensitivity, and color weakness) accounted for a substantial amount of the variance in Stroop performance [77]. The preceding findings suggest that impairments in the early perceptual-encoding stage of stimulus color contribute to slow performance on neuropsychological tasks requiring speeded naming of color, as well as conceptual and attentional factors [76].

Stimulant-induced increases in the availability of dopamine would be expected to be reflected in retinal dopaminergic tone and thus have therapeutic effects on the short-wavelength mechanism. Thus, methylphenidate would be expected to improve visual functioning, including the speed of color processing and naming. Indeed, there is preliminary evidence of beneficial effects of methylphenidate on color naming in children with ADHD [8,23].

### Gender effects

Alterations in short-wavelengths mechanism may be gender-related. Notably, estrogen has been found to have a modulatory influence on dopamine activity [78] and a few studies have revealed significant variations in dopaminergic tone and dopamine receptor density that are sex-specific [79,80]. Correspondingly, gender-related differences observed in visual-cortical fMRI BOLD response to blue light (higher BOLD signal change in males), but not to red light, have been reported that may be related to variations in dopamine function and/or the effects of estrogen on dopamine [81]. Furthermore, visual pattern reversal evoked potentials have been found to vary with menstrual phase in females and display faster conduction times during the period of peak estrogen levels [82]. Given the incidence of ADHD is estimated to be three times greater in males than females, the relationship between estrogen and dopamine may provide an important area for further investigation.

### Developmental effects

The limited data available on developmental changes in retinal and central dopaminergic mechanisms, color perception, and color naming, suggest that the retinal dopamine hypothesis will likely hold for children, adolescents, and adults with ADHD. For example, animal research indicates that dopaminergic neurons are among the first neurochemical systems to appear in the developing retina and that the neural retina and dopaminergic system interact closely in a two-way manner throughout developmental period [53]. It is only as the animal passes through maturity towards senescence that the number of retinal dopamine neurons decrease [53]. Thus, assuming that hyperdopaminergic function in ADHD occurs early in pre- or post-natal life, parallel hypofunctioning of the retinal dopamine system is expected to have a detrimental effect on the development of blue-yellow color vision and these early deficits are likely to persist.

On the other hand, the increasing efficiency of the mature (adult) brain permits the development and use of compensatory strategies. Thus, adults with ADHD, although impaired in color perception relative to healthy peers, may be able to call upon compensatory attentional strategies to enhance performance on tasks requiring rapid perception of blue and yellow stimuli, thereby exhibiting better performance than children or adolescents with ADHD.

## Testing the hypothesis

Three strategies are proposed to provide a rigorous test of the hypothesis: 1) direct assessment of color vision pathways in individuals with ADHD versus a comparison group of healthy peers; 2) evaluation of the relationship between color vision (particularly for blue-yellow stimuli) and performance on tasks requiring speeded color processing; and 3) investigation of stimulant effects on both color vision and performance on those tasks in individuals with ADHD. Color vision can be assessed directly using clinical measures that are sensitive to problems with stimuli along the blue-yellow axis [83-85] as well as using color visual evoked potential (VEP), which is an objective, sensitive, and non-invasive measure of neuronal integrity of red-green and blue-yellow pathways [86-88]. A detailed neuro-opthalmological examination is also required to rule out confounding factors, such as problems with visual acuity, refraction, contrast sensitivity, or structure of the fundus (ocular media, posterior pole, and macular area of the retina). Evidence of selective impairments in blue-yellow but not red-green color perception would mitigate an alternative explanation that impaired perception of stimulus color reflects an attention dysfunction, and be consistent with the proposed hypothesis that dysregulation of central and retinal dopamine impairs blue-yellow color perception. Next, regression techniques are required to examine the relationship between performance on various standardized neuropsychological tests requiring speeded color processing [30,31,89] and the color vision measures (e.g., VEP latencies, continuous scores from clinical color vision tests). Finally, a randomized, double-blind, placebo-controlled, within-subject design is required to test whether stimulant-induced increases in central dopamine also produces parallel increases in retinal dopamine, as indexed by improved performance on both color vision and speeded color naming.

## Theoretical and Clinical Implications of the Hypothesis

Convergent findings from empirical investigations and neural network simulation support the hypothesis that color perception contributes to performance on neuropsychological tasks requiring speeded color naming [70,71]. Preliminary support for our hypothesis that color perception of blue-yellow (but not red-green) stimuli is impaired in ADHD as a result of deficient central and retinal dopamine is provided by findings from our recent small-scaled study that demonstrated selective impairments in blue-yellow (i.e, not red-green) color perception in children with ADHD [83]. Also, the contrasting effects of dopaminergic and noradrenergic drugs on color naming in individuals with ADHD [8,23,91] are consistent with the premise that increases in central dopamine will also increase retinal dopamine, resulting in improved color processing and naming.

If substantiated in larger well-controlled studies, evidence that blue-yellow color perception problems contribute to poor performance on neuropsychological tasks of executive function requiring speeded color processing, would necessitate careful consideration to stimulus color when interpreting performance on many of the standard neuropsychological tests. Also, evidence of specific impairments in blue-yellow color perception (and other visual functions) would necessitate a reconsideration of current neuropsychological models of ADHD, which posit the core deficits to be in higher-order executive functioning and not at the level of sensory and perceptual processing. From a clinical perspective, this retinal-dopaminergic hypothesis might indicate the need to include a visual examination in the assessment of ADHD, and raises the possibility that electroretinogram blue cone amplitudes may also be a possible neurobiologic marker related to central dopamine function in ADHD, as well as in cocaine-dependent patients [67].

## List of Abbreviations

ADHD: Attention-Deficit/Hyperactivity Disorder

RAN: Rapid Automatized Naming Test

VEP: Visual evoked potential

## Competing interests

The author(s) declare that they have no competing interests.

## Authors' contributions

RT developed the hypothesis and prepared the initial draft of the manuscript; TB refined it and conducted a preliminary test of it; DG conducted an in-depth literature search. All authors helped revising the manuscript and have read and approved the final manuscript.
